# Robotic arm-assisted total knee arthroplasty improves preoperative planning and intraoperative decision-making

**DOI:** 10.1186/s13018-021-02815-6

**Published:** 2021-11-15

**Authors:** Xufeng Wan, Qiang Su, Duan Wang, Mingcheng Yuan, Yahao Lai, Hong Xu, Zongke Zhou

**Affiliations:** Department of Orthopedics, West China Hospital, Sichuan University, 37# Wuhou Guoxue Road, Chengdu, 610041 People’s Republic of China

**Keywords:** Total knee arthroplasty, Robot-assisted surgery, Preoperative TKA planning, TKA implant sizing

## Abstract

**Background:**

The reliability of robotic arm-assisted total knee arthroplasty (RA-TKA) has been previously reported. In this study, we evaluated the predictive accuracy of the RA-TKA system in determining the required bone resection and implant size preoperatively and its effect on intraoperative decision-making.

**Methods:**

Data on the outcomes of RA-TKA procedures performed in our department were prospectively collected. A three-dimensional model of the femur, tibia, and fibula was reconstructed using standard computed tomography (CT) images. The model was used preoperatively to predict bone required resection for the femur and tibia and implant size. Intraoperatively, the images were registered to the local anatomy to create a patient-specific model for decision-making, including real-time measurement of the medial-to-lateral difference in the extension/flexion gap and TKA component alignment. Differences between predicted and real bone resections and implant size were evaluated, and the post-TKA mechanical axis of the lower limb and difference in medial-to-lateral flexion/extension gap were measured.

**Results:**

The analysis was based on the data of 28 patients who underwent TKA to treat severe osteoarthritis. The RA-TKA system successfully predicted the femoral and tibial component within one implant size in 28/28 cases (100%). For the 168 bone resections performed, including both femoral and tibial cuts, the resection was within 1 mm of the predicted value in 120/168 (71%) of the cuts. The actual versus predicted bone resection was statistically different only for the lateral tibial plateau (*p* = 0.018). The medial-to-lateral gap difference was between − 1 and 1 mm, except in one case. The achieved lower limb alignment was accurate overall, with the alignment being within < 1.0° of the neutral mechanical axis in 13/28 cases (46%) and within < 3.0° in 28/28 cases (100%).

**Conclusions:**

The RA-TKA system provided considerable pre- and intraoperative surgical assistance to achieve accurate bone resection, appropriate component sizing, and postoperative alignment after RA-TKA.

## Introduction

Total knee arthroplasty (TKA) is the standard treatment for patients with end-stage knee disease, with the annual number of TKA procedures expected to increase to 1.26 million by 2030 in the USA alone [1]. Component malalignment, however, is associated with an increased rate of polyethylene wear, resulting in a shorter survival rate after TKA compared to the wear and survival rates of TKAs with a neutral alignment [2]. Currently, intramedullary femoral and extramedullary tibial guides are generally used to achieve coronal component alignment; however, surgeons may not always achieve the bone resection necessary for an optimal alignment [3–5]. Inappropriate bone resection and associated flexion/extension gaps can result in suboptimal TKA component positioning, leading to component malalignment [6, 7]. Surgical error leading to failed TKA can be avoided [8, 9], and therefore, focus has been placed on developing strategies to improve the accuracy of implant sizing and positioning to recreate the knee joint line and to appropriately balance soft tissue to achieve an accurate overall implant and lower limb alignment [10, 11]. In this regard, robotic arm-assisted TKA (RA-TKA) has emerged as a reliable method to achieve accurate and precise lower limb alignment after TKA [10, 11]. Based on three-dimensional (3D) computed tomography (CT), the robotic arm-assisted system is designed to minimize the margin of error associated with bone resection and to provide real-time guidance for intraoperative TKA component positioning prior to final implantation. Moreover, the system provides the ability to accurately predict implant size preoperatively, which can improve operative efficiency [12], although this has not been comprehensively evaluated. Accordingly, the aim of our study was to evaluate the predictive accuracy of the RA-TKA system in determining the required bone resection and implant size preoperatively and its effect on intraoperative decision-making. We hypothesized that the RA-TKA system would provide accurate prediction of bone resection and component size and would guide accurate component alignment intraoperatively.

## Methods

This study was approved by the Ethics Committee for Clinical Trials of the department (HX-IRB-AF-19-V4.0). This study was registered in the Clinical Trial Registry (ChiCTR2100042933). Data regarding robot-assisted surgery performed in our unit were prospectively collected. All RA-TKAs procedures were performed using a single robot-assisted surgical system (YUANHUA-TKA) by a single surgeon. All patients provided consent for surgery and to have their data included in this study.

### Preoperative planning

**Fig. 1:**
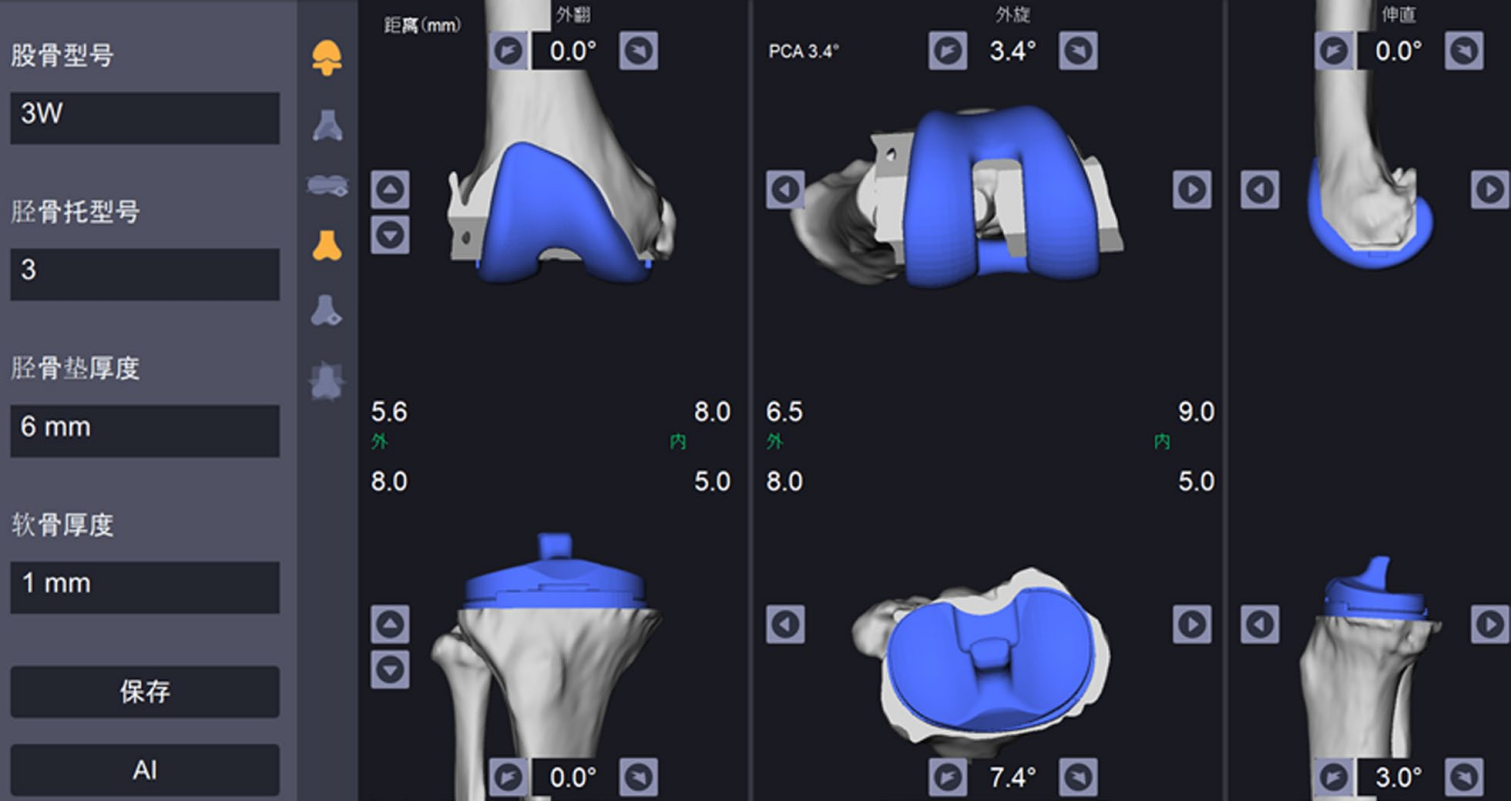
Computer images of planning for robot-assisted total knee arthroplasty with three-dimensionally reconstructed tibial and femoral component sizes 股骨型号, size of femoral implant; 胫骨托型号, size of tibial plateau; 胫骨垫厚度, Tibial cushion thickness; 软骨厚度, cartilage thickness; 外翻, valgus; 外旋, external rotation; 伸直, extension; 外, lateral; 内, medial

For preoperative planning, standard CT of the hip, knee, and ankle regions was acquired prior to the surgery, according to the manufacturer’s protocol. The axial CT images were exported to a CD in DICOM file format, segmented, and stored to a dedicated laptop, with a proprietary operating system. A 3D model of the patient's femur, tibia, and fibula was generated from segmented CT images and anatomical bone landmarks selected for each bone to establish a local reference system. These images were then registered to the robotic system to create a virtual operative plan. Achieving a neutral mechanical axis of the lower limb was the target implant position, with the required planned bone resections and implant size needed predicted using the RA-TKA system. These preoperative renderings were used by the robotic system to map an intraoperative plan, including the location of the tibial and femoral cuts and, based on this information, the tibial and femoral implant component sizes to be used. (Fig. 1).

### Robotic arm-assisted surgery

Intraoperatively, the RA-TKA system does not require the use of cutting blocks or alignment guides and allows for tracking of the position of the femur and tibia. Selected bone landmarks were registered intraoperatively using the robotic probe. The robotic software was then used to generate a patient-specific model for real-time updating of the operative plan. Bone cuts were then performed using the RA-TKA system, based on the preoperative plan, in the following order: proximal tibia, anterior condyle, posterior condyle, posterior chamfer, distal femur, and anterior chamfer. The final bone resections were measured using a previously reported protocol [13].

Using the robotic software, pre- to post-bone cut extension gaps were measured, and the medial-to-lateral difference in flexion/extension gaps was calculated. After installing the test die, the robot system can calculate the medial and lateral gaps by keeping the flexion or extension position, and the difference between the medial-to-lateral gaps difference can reflect the medial/lateral laxity. An appropriately balanced knee was defined by a medial-to-lateral flexion/extension gaps difference of < 2 mm. The robot-predicted implant size was compared to the final implant size used. Postoperative alignment was measured by the hip-knee-ankle angle (HKA) in full-length X-ray of both lower extremities.

### Statistical analysis

The following outcomes were evaluated: difference between the planned and final bone resections and coronal limb alignment of the knee; the absolute medial-to-lateral difference in flexion/extension gaps; predicted and actual bone resections; the difference between predicted and final intraoperative flexion/extension gaps; and the postoperative alignment. We also compared the implant size predicted preoperatively, using the RA-TKA system, to the actual implant size used. Predicted and postoperative measures were compared using a paired-samples t test, with significance set at a *p* value < 0.05. All patient data were recorded using Microsoft Excel, with the statistical analysis performed using SPSS Statistics (version 24; SPSS, Chicago, IL, USA).

## Results

The study included 28 patients who underwent TKA for the treatment of severe osteoarthritis: mean age, 65 (standard deviation (SD), 6.4) years and mean body mass index (BMI) of 27.4 (SD, 3.0) kg/m^2^. Details regarding the sex distribution and side operated are shown in Table 1.

**Table 1:** Demographics

Age, years (mean ± SD)	65.2 ± 6.4
Gender, N (%)	
Female	20 (71%)
Male	8 (29%)
BMI, (mean ± SD)	27.4 ± 3.0
Operative side, N (%)	
Left	17 (61%)
Right	11 (39%)
Diagnosis	Osteoarthritis

BMI, body mass index

### Implant size

The accuracy of the predicted implant size is presented in Table 2. The size of the femoral component was successfully predicted using the RA-TKA in 26/28 (93%) cases, with the size predicted within one implant size in all cases (100%). The implant size of the tibial component was successfully predicted in 25/28 cases (89%). Among the cases not accurately predicted, the size was predicted within one implant size in 28/28 of cases (100%).

**Table 2:** Implant size, actual versus RA-TKA predicted

Size differential (implant)	0	1
Femoral implant	26 (93%)	2 (7%)
Tibial implant	25 (89%)	3 (11%)

### Bone resection

**Fig. 2:**
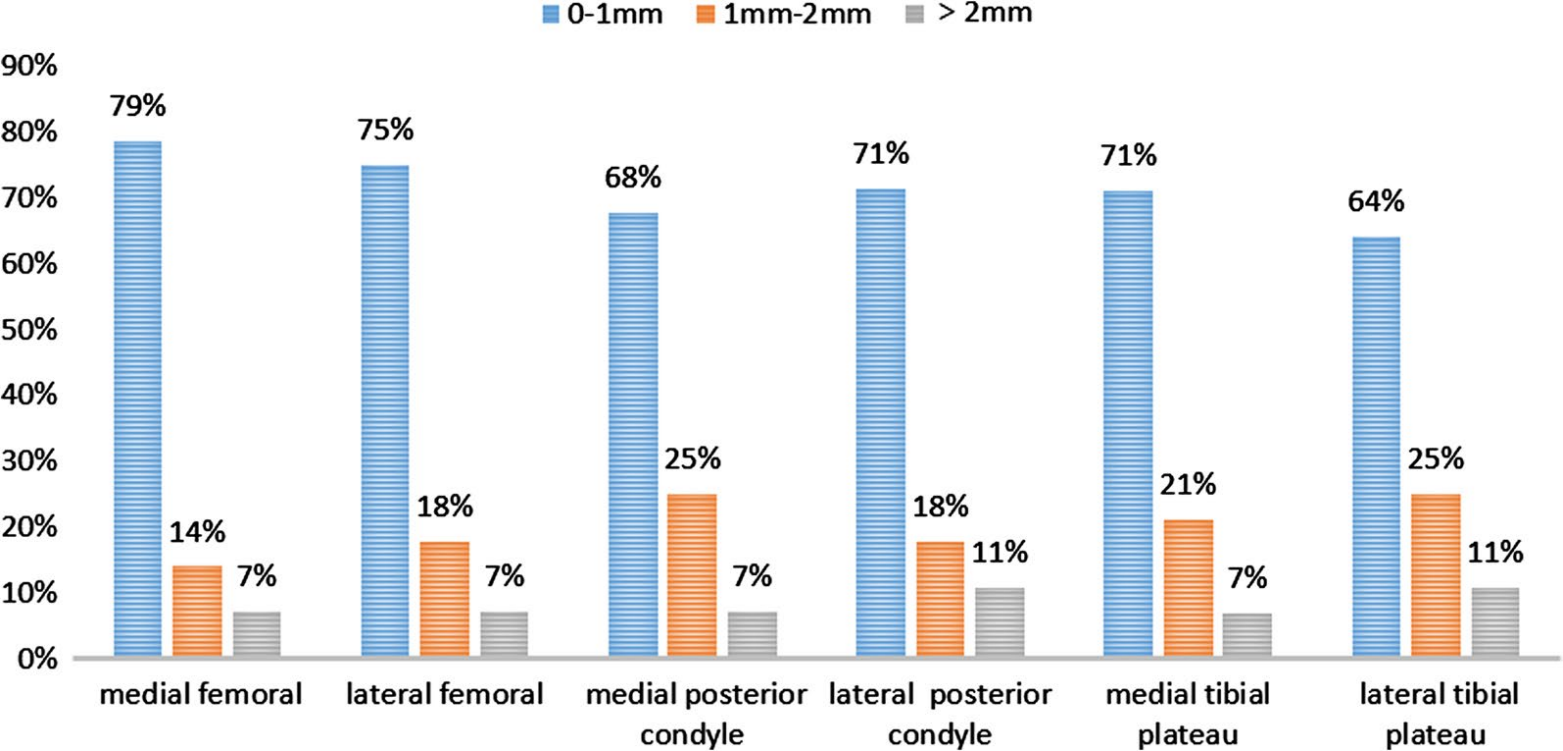
The distribution of absolute difference between the preoperative prediction and the actual bone resection

The mean absolute difference between the predicted and actual medial and lateral bone resection cuts are reported in Table 3. For the femur, there was no difference between the predicted and actual medial and lateral resections, respectively, as follows: distal femoral resection, 1.26 ± 0.83 mm and 1.25 ± 0.91 mm; posterior femoral condyle resection, 0.81 ± 0.57 mm and 0.90 ± 0.74 mm. For the tibia, while there was no difference for the medial tibial plateau resection, 1.14 ± 0.88 mm, the difference between predicted and actual resection of the lateral tibial plateau was significant (*p* = 0.018). Overall, of the 168 bone resections performed, the difference between the predicted and actual cuts was < 1 mm in 120 (71.43%). The distribution of absolute differences between preoperative predicted and actual bone resection is shown in Fig. 2.

**Table 3:** Summary of the actual bone resection compared to the predicted

Location	Predicted resection (mm) mean (SD)	Actual resection (mm) mean (SD)	Absolute difference mean (SD)	*p* value
Medial femoral	8.78 (0.60)	8.71 (1.51)	1.26 (0.83)	0.82
Lateral femoral	7.83 (1.57)	8.75 (2.06)	1.25 (0.91)	0.07
Medial posterior condyle	9.61 (1.06)	9.79 (1.51)	0.81 (0.57)	0.61
Lateral posterior condyle	6.96 (1.10)	7.02 (1.50)	0.90 (0.74)	0.86
Medial tibial plateau	4.9 (1.51)	4.89 (2.00)	1.14 (0.88)	0.99
Lateral tibial plateau	8.79 (0.42)	9.43 (1.33)	1.06 (0.85)	0.02

### Measurement flexion/extension gaps

After completion of bone resections, the medial-to-lateral difference in the extension gap was between − 1 and 1 mm (mean, 0.556 mm; SD, 0.511 mm), as was the medial-to-lateral difference in flexion gap (mean, 0.778 mm; SD, 0.647 mm), except for one case in which the difference was > 1 mm (Table 4).

**Table 4:** Absolute medial-to-lateral difference in the extension and flexion gap, pre-versus post-resection

Location	Pre-bone resection	Post-bone resection	*p* value
Extension	2.56 (1.97)	0.56 (0.51)	< 0.0001
Flexion	1.67 (0.69)	0.78 (0.65)	< 0.0001

### Postoperative limb alignment

**Fig. 3:**
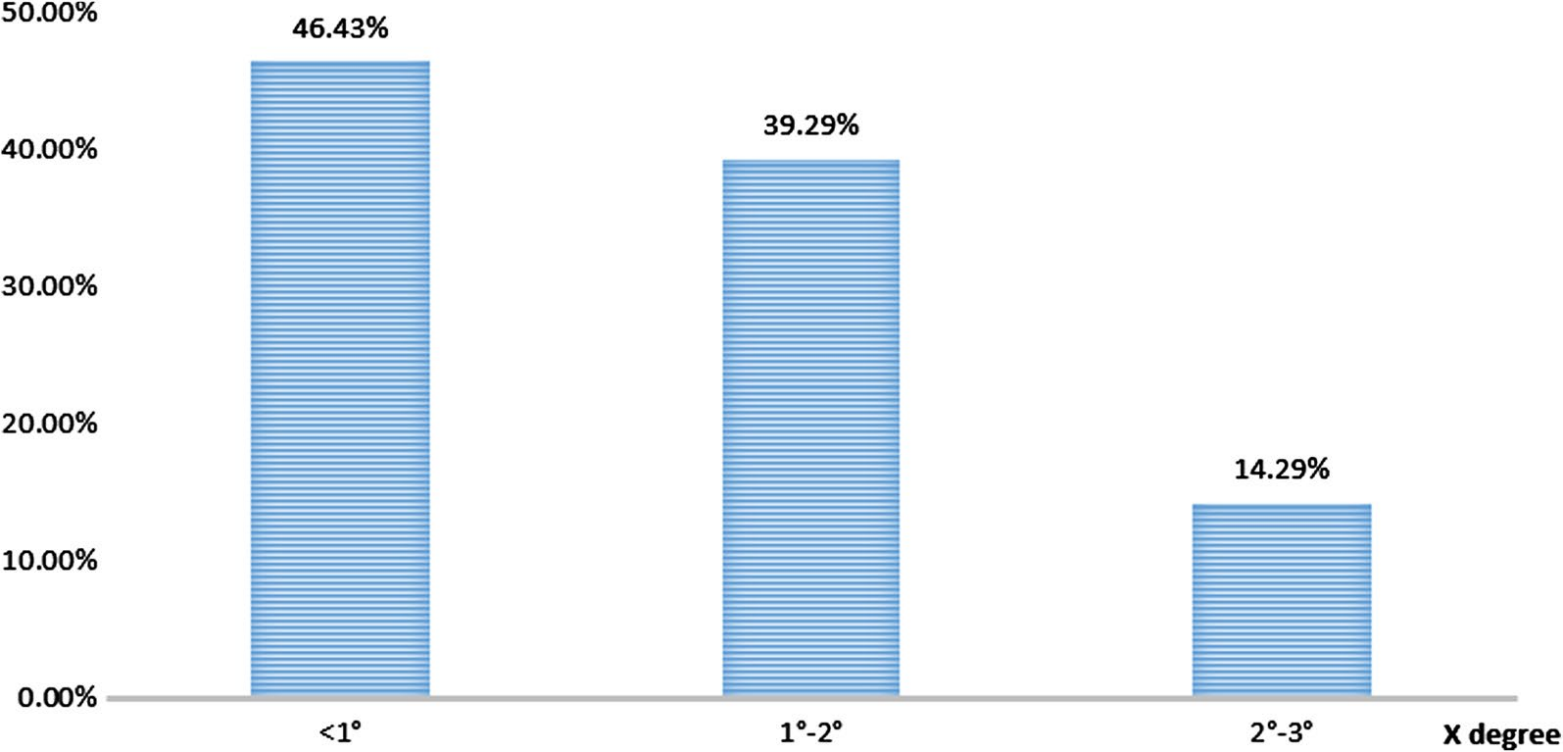
Distribution of deviation in postoperative limb coronal alignment from the neutral mechanical axis of the lower limb

The mean absolute difference between the final limb coronal alignment and the neutral mechanical axis (180°) of the lower was 1.13° (SD, 0.61°), with the alignment being < 1.0° in 13/28 cases (46%) and < 3.0° in 28/28 cases (100%). The distribution of post-TKA coronal plane alignment is shown in Fig. 3.

## Discussion

The current study supports the use of the RA-TKA system for accurate preoperative planning with regard to bone resection and implant size, intraoperative flexion/extension gap and postoperative alignment. As far as we know, this is the first report on preoperative prediction and intraoperative adjustment of Chinese-made robot.

This is consistent with previous research reporting the benefits of RA-TKA in achieving an optimal coronal plan alignment, better clinical and functional outcomes, and soft tissue protection [14–16]. In our study, the precision of the alignment using the RA-TKA system was high overall, with the post-TKA alignment being within 1.13° of the neutral mechanical axis, on average, with an SD of 0.61°. This accuracy in post-TKA alignment when using the RA-TKA is consistent with previous findings [17–19]. In previous reports of 31 [17] and 261 [14] RA-TKA cases, no malalignment > 3° was noted. A systematic review by Mannan et al. [20] reported a higher accuracy in post-TKA alignment for RA-TKA than the conventional TKA method. The higher accuracy in lower limb alignment achieved with RA-TKA reflects the accuracy in preoperative prediction and intraoperative measures of bone resections, flexion/extension gaps, and implant component alignment.

In our study, the difference between predicted and actual femoral and tibial bone resection was within 1 mm in 71% of our cases. We do note that the accuracy of predicted resections was better for the femur than the tibia, although this difference in accuracy was minimal. Furthermore, the precision of bone resection was high for all six location of resections, with an overall SD of < 1.0 mm. Our findings are consistent with those of Sires et al. [11] who reported a high accuracy for both femoral and tibial bone resections, with 94% of actual cuts being < 1 mm of the predicted cuts. We note that the SD values of the precision of the predicted resections were lower for Sires et al. (0.32 mm and 0.30 mm for the distal femur cut and tibia cut, respectively) than ours. Differences in accuracy and precision measures between our study and those of Sires et al. can be explained by the following factors. Foremost, Sires et al. reported on anterior femoral and tibial cuts while we included posterior femoral cuts as well. We also used Vernier calipers to measure bone resections intraoperatively. Although Vernier calipers have been previously used during TKAs, there is some measurement error compared to computer/image-based measurements, although this measurement error is typically < 1 mm and therefore might be negligible [11, 13]. Our predictions were based on preoperative CT and, therefore, did not take into account any intraoperative adjustments or manipulations. Moreover, we considered a cartilage thickness of 1 mm in our preoperative planning, which was adjusted intraoperatively based on the actual cartilage thickness. In fact, the greater actual bone resection of the lateral tibial plateau than predicted preoperatively likely reflects greater thickness of peripheral cartilage measured intraoperatively.

Accuracy of the resection of the distal femur and proximal tibia is important to set the limb alignment at the knee which, ultimately, will affect tension of the knee ligaments in extension. We found that for our study group, the RA-TKA system provided substantive assistance to the surgeon in minimizing the flexion/extension gaps and achieving a more balanced knee both intra- and postoperatively. In fact, the medial-to-lateral flexion/extension gap after bone resection was < 1 mm, except in one. This is comparable to the findings of Marchand et al. [21] who reported a flexion gap difference after bone resection of − 2 to 2 mm (mean, 0 mm) in 99% of their 332 cases of TKA and an extension gap of − 1 to 1 mm (mean, 0 mm). Balanced gaps have been considered a prerequisite for good function and endurance in TKA, and a balanced knee could affect clinical outcomes [22]. Moreover, this precision and intraoperative feedback on the gap balancing could possibly minimize limitations in the conventional techniques and sizing options [23].

An important finding of our study was the accurate prediction of the implant size, resulting from accurate bone resection and balancing of the flexion/extension gaps. It has been previously reported that variation in the size and shape of the distal femur may result in poor accuracy of TKA alignment [24, 25]. Our accurate sizing was consistent with the findings of Marchand et al. [21, 24] who reported that the RA-TKA system accurately predicted both femoral and tibial component sizes in 98% of cases. This reliability in prediction of implant size is critical in large academic and community centers, allowing for quality control and more accurate inventory management, ensuring that the correct implant is available prior to the surgery. In the operative theater, knowing the size of the implant before surgery reduces the number of surgical trays needed, which improves the setup time and operative resources needed, [12, 21, 26] as well as reduce operative time and costs [12]. Prediction of prosthesis size has been explored using conventional templates, computer navigation, patient-specific instrumentation, and robotic-assisted arthroplasty techniques. Many of these methods have proven to not be highly accurate [27]. As an example, the accuracy of the template technique has been reported to range between 28 and 48% for the femoral component and between 37 and 55% for the tibial component [27]. Iorio et al. [28] reported an accuracy of CT-based prediction of 93% for the femoral component and 54% for the tibial component, which were better than the accuracy rates for 2D digital templating. A preoperative accuracy of 97% for predicting the size of the femoral component and 93% for the tibial component allows the surgical team to confidently reduce the number of prostheses of different sizes opened on trays [29, 30]. TKA component overhang is responsible for 27% of all cases of significant knee pain after TKA [31]. Preoperative planning based on 3D CT image reconstruction, as we used, can better assist doctors in selecting the appropriate prosthesis and obtaining an optimal implant positioning.

The limitations of our study need to be acknowledged in the interpretation of our findings for practice. First, the sample size was small. However, our SD measures were low, overall, indicating the repeatability of our findings. Notwithstanding this repeatability, a larger cohort study would provide a more definite conclusion. Second, only patients who underwent an RA-TKA procedure were included; it would be beneficial to compare outcomes of the RA-TKA procedure to other methods. This was a single site study with all TKAs performed by a single surgeon. Third, although this allowed us to control for potential confounding factors, such as between-surgeon factors, it does limit the generalizability of our findings. Finally, there is a need to evaluate patient-related clinical outcomes.

## Conclusion

The RA-TKA system allowing for 3D manipulation, visualization, and planning using 3D CT image reconstruction as inputs, provides an accurate prediction of the TKA procedures and assisted the surgeon in achieving accurate and reproducible bone resection, component position, balancing of the flexion/extension gaps, and postoperative alignment. RA-TKA surgery is a trend in the development of intelligent systems for orthopedic surgery, which can improve surgeons’ confidence and accuracy.

## Data Availability

The data sets used and/or analyzed during the current study are fully available on reasonable request.

## Abbreviations

RA-TKA: Robotic arm-assisted total knee arthroplasty
CT: Computed tomography
TKA: Total knee arthroplasty
3D: Three-dimensional
SD: Standard deviation
BMI: Body mass index
M: Mean
N: Number
